# Bioinformatics Identification and Experimental Validation of Ferroptosis- and Immune Infiltration-Associated Biomarkers in Ischemic Stroke

**DOI:** 10.3390/cimb47121066

**Published:** 2025-12-18

**Authors:** Fan Huang, Mingjing Zhu, Huihui Wang, Zilong Du, Qianqian Wu, Yongjing He, Yilin Liang, Wanxiang Hu, Lu Xie

**Affiliations:** 1 Department of Physiology, Pre-Clinical Science, Guangxi Medical University, Nanning 530021, China; 2 Department of Radiation Oncology, the Affiliated Tumor Hospital of Guangxi Medical University, Nanning 530021, China

**Keywords:** ischemic stroke, cerebral ischemia–reperfusion, ferroptosis, immune infiltration

## Abstract

Ischemic stroke (IS) continues to pose a significant threat to human health. Few studies have explored the connection between ferroptosis-related genes and immune infiltration in the context of IS. Initially, 303 differentially expressed genes were identified, from which four characteristic genes were distinguished, all validated for their excellent diagnostic efficacy. Animal experiments confirmed significant brain injury and Ferroptosis post-ischemia–reperfusion in rats, with increased expression of Sdcbp, Ppia, and Sec61g, but no change in Rpl22. Furthermore, these key genes were closely associated with levels of immune infiltration. Notably, Rpl22 and Ppia were regulated by nine common transcription factors. Sdcbp and Rpl22 were most abundantly expressed in Microglia, and Ppia in Oligodendrocytes, while Sec61g exhibited lower overall expression, all showing high activity in immune metabolic pathways. Bioinformatics analysis and experimental verification indicate that Sdcbp, Ppia, and Sec61g are associated with ferroptosis and immune infiltration in IS, and hold promise as therapeutic targets for IS treatment.

## 1. Introduction

Stroke ranks as the second leading cause of death and disability worldwide [1], with its incidence continually rising due to shifts in population demographics and increasing rates of diabetes [2] and obesity [3]. The cornerstone of ischemic stroke (IS) management is the restoration of blood perfusion to salvage jeopardized neurons [4]. However, survivors often endure significant challenges posed by reperfusion injuries [5], known as cerebral ischemia–reperfusion injury (CIRI). Despite the availability of treatments like intravenous thrombolysis (IVT) [6], endovascular thrombectomy (EVT), and antithrombotic therapies, the reperfusion rates are suboptimal, the therapeutic window is narrow, and contraindications to thrombolysis exist [7], benefiting only a minority of patients. Thus, identifying potential biomarkers and exploring the molecular mechanisms of CIRI to discover novel therapeutic targets have become imperative.

The extent of damage in IS largely depends on the number of neurons that succumb to ischemia-related death in the affected brain regions. Iron-dependent cell death, or ferroptosis, first described by Dixon et al. in 2012 [8], represents a regulated cell death modality characterized by iron accumulation and lipid peroxidation, distinct from apoptosis, necrosis, autophagy, and other forms of cell death. Mechanistically, unsaturated fatty acids on the cell membrane undergo lipid peroxidation under the action of divalent iron or lipoxygenases, leading to cell demise, while simultaneously downregulating the antioxidant glutathione system’s core enzyme Gpx4 [9]. Post-ischemic observations have noted lipid peroxidation and iron accumulation along with decreased GPX4 expression, elevated levels of ACSL4, COX2, and ROS [10,11,12]. Additionally, the ferroptosis inhibitors ferrostatin-1 and liproxstatin-1 have demonstrated neuroprotective effects in mice subjected to middle cerebral artery occlusion (MCAO) [13]. These findings underscore the involvement of ferroptosis in neuronal damage following IS. The increased permeability and compromised integrity of the blood–brain barrier post-ischemia facilitate peripheral T-cell invasion and infiltration [14], which promote leukocyte adhesion to the cerebrovascular system, triggering thrombo-inflammatory responses that exacerbate IS damage [15]. Thus, monitoring brain immune and inflammatory responses can guide clinical decision-making [16]. Ferroptosis, as a mode of inflammatory cell death, is associated with the release of pro-inflammatory mediators and lipid peroxides [17]. Accumulating evidence indicates that cells undergoing ferroptosis release specific damage-associated molecular patterns (DAMPs) [18]. During cerebral ischemia–reperfusion, these DAMPs activate pattern recognition receptors—such as Toll-like receptor 4 (TLR4) and the NLRP3 inflammasome—in immune cells, thereby initiating a robust inflammatory response that exacerbates brain injury [19]. This self-perpetuating “ferroptosis–immunity” vicious cycle represents a highly attractive therapeutic target. Conventional neuroprotective strategies have primarily focused on directly rescuing vulnerable neurons, yet their clinical translation has been hampered by a narrow therapeutic time window and limited target diversity. In contrast, targeting the role of ferroptosis in driving immune infiltration offers a complementary therapeutic approach. By inhibiting ferroptosis, it may be possible not only to reduce primary neuronal loss directly but also to attenuate the release of pro-inflammatory DAMPs at their source. This dual mechanism would mitigate excessive neuroinflammation, thereby preserving the salvageable penumbra and potentially improving long-term functional outcomes. Therefore, elucidating the crosstalk between ferroptosis and neuroinflammation not only reveals a novel pathological dimension of ischemic brain injury but also opens avenues for developing integrated treatment strategies that combine neuroprotection with immunomodulation.

**Figure 1:**
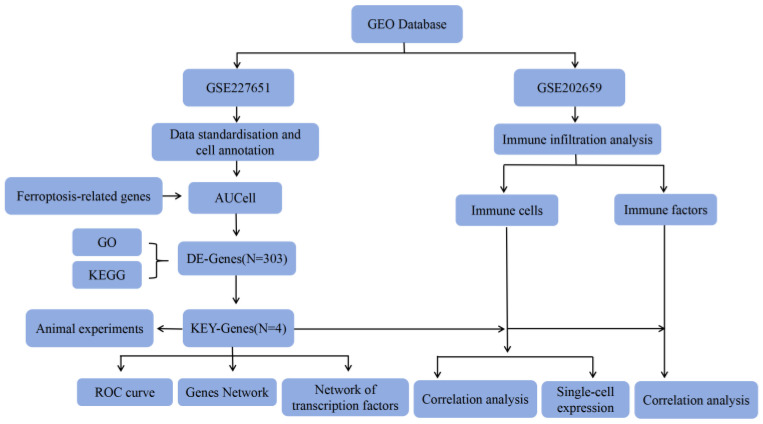
Workflow of the study.

The rapid development of genomic microarrays and high-throughput sequencing technologies has made the exploration of biomarkers and molecular mechanisms of IS increasingly popular through bioinformatics analyses. This study employs single-cell sequencing techniques to identify key genes through differential analysis and machine learning algorithms, validating them through correlation analyses, ROC curves, interaction networks, and efficacy diagnostics. Ultimately, it examines the connections between key genes and the level of immune cell infiltration, regulation by transcription factors, expression in immune metabolic pathways, and interactions with disease-related genes (Figure 1).

## 2. Materials and Methods

### 2.1. Data Acquisition

The Gene Expression Omnibus (GEO) database (https://www.ncbi.nlm.nih.gov/geo/info/datasets.html, accessed on 23 June 2024), managed by the National Center for Biotechnology Information (NCBI), serves as a repository for gene expression data. We downloaded the Series Matrix File for GSE202659, which includes six expression profiles, with three from the control group and three from the disease group, annotated under GPL24247. Additionally, single-cell data files for GSE227651 were obtained, encompassing four samples with complete single-cell expression profiles—comprising one control and three disease cases.

### 2.2. Quality Control

We initiated quality control by loading the expression profiles using the Seurat v4 package. Cells were filtered based on total UMI counts, the number of expressed genes, and the proportions of mitochondrial and ribosomal gene expressions. Cells exhibiting high percentages of mitochondrial or ribosomal expressions—indicative of impending cell death—were identified. Quality control was further refined using the Median Absolute Deviation (MAD); values deviating more than three MADs (nCount_RNA, nFeature_RNA, percent.mt) from the median were considered outliers and excluded. DoubletFinder (V2.0.4) was then employed to remove doublets from each sample, thus concluding the cell quality control process.

### 2.3. Data Normalization

Global normalization was conducted using the LogNormalize method, scaling each cell’s total expression to 10,000 before log transformation. Cell cycle scores were computed using the CellCycleScoring function, and highly variable genes were identified with FindVariableFeatures. The ScaleData function was used to adjust for variations in gene expression due to mitochondrial content, ribosomal content, and cell cycle differences. PCA was performed on the expression matrix to reduce dimensionality, and batch effects were corrected using Harmony. Dimensionality was further reduced nonlinearly using the RunUMAP function.

### 2.4. Enrichment Analysis

Functional annotations of marker genes were conducted using the Metascape database (www.metascape.org, accessed on 3 July 2024) to explore their functional correlations extensively. Gene ontology pathway analysis was performed, considering a minimum overlap of three and a *p*-value of ≤0.01 as statistically significant.

### 2.5. Lasso Regression for Feature Selection

LASSO, a regularization technique that enhances model simplicity and interprets co-linearity by shrinking some coefficients towards zero, was applied using the glmnet v4.2.2 package. This method was specifically used for selecting features that serve as diagnostic biomarkers for the disease.

### 2.6. Immune Infiltration Analysis

The CIBERSORT method, based on support vector regression, was applied to assess immune cell types in the microenvironment using the expression matrix to deconvolute 547 biomarkers across 25 immune cell phenotypes, including T cells, B cells, plasma cells, and myeloid subgroups. Pearson correlation analysis was performed on the dataset GSE202659 to infer the relative proportions of 25 infiltrating immune cells and correlate these with gene expression levels.

### 2.7. Transcription Factor Regulatory Networks

The RcisTarget v4.3.1 package was used to predict transcription factors based on motifs. All calculations were motif-based, and the normalized enrichment scores (NES) depended on the total number of motifs in the database. In addition to annotated motifs, further annotations were inferred based on motif similarity and gene sequences. The first step in estimating motif overexpression involved calculating the Area Under the Curve (AUC) for each motif-gene set pair based on recovery curves sorted by gene sets. Each motif’s NES was calculated based on the distribution of AUCs among all motifs in the gene set.

### 2.8. Key Gene Expression, Immune Metabolic Pathways, and Disease-Related Co-Expression Networks

Key gene expressions in single cells were visualized using Dotplot and FeaturePlot functions in the Seurat v4 package. AUCell was used to quantify immune metabolic pathway genes in single cells, and bubble charts were employed to display the expression variations in key genes related to immune metabolic pathways. Disease-related genes were obtained from the GeneCards database (https://www.genecards.org/, accessed on 15 September 2024), and those with the highest relevance scores were selected for co-expression network analysis through correlation analysis.

### 2.9. Animal Experiments and MCAO/R Rat Model

Twenty-eight adult male Sprague-Dawley rats (200–240 g) were acquired from the animal experimental center of Guangxi Medical University, with a license number SCXK (Gui) 2020-0003. The animals were housed under controlled conditions (23 ± 2 °C) with ad libitum access to food and water. The MCAO/R model was established using the thread embolism method [20]. Rats were anesthetized with 2% pentobarbital sodium (50 mg/kg), fixed in a supine position on an operation board, and underwent a longitudinal incision on the midline of the neck after depilation and disinfection. The common carotid artery, internal carotid artery, and external carotid artery were bluntly separated, with the external artery ligated. An incision was made on the common carotid artery 5 mm from the internal carotid intersection, and a thread was inserted until slight resistance was felt. The thread was then secured to prevent slippage, and the incision was sutured. After 2 h of ischemia, the embolus was removed to restore blood flow. After 24 h of reperfusion, the Zea Longa scoring method [21] was used to assess the MCAO/R model rats. Rats scored 2 or higher were considered successfully modeled. The scoring criteria were as follows: 0 points for no neurological impairment; 1 point for incomplete extension of the contralateral forelimb; 2 points for circling to the contralateral side; 3 points for falling to the contralateral side; 4 points for loss of spontaneous walking and consciousness.

### 2.10. H&E Staining

Brain tissue morphology was observed through H&E staining. After perfusion for 24 h, the brain was removed and soaked in 4% polyformaldehyde overnight. It was then dehydrated through a gradient ethanol series in a dehydration machine as follows: 75% ethanol for 4 h, 85% for 2 h, 90% for 2 h, 95% for 1 h, 100% for 1 h, and xylene for 15 min. Use a slicer to slice coronal sections of 4 µm at approximately −2.0 mm to −3.0 mm posterior to bregma, and then dewaxed in xylene for 10 min and rehydrated in a descending series of ethanol concentrations before being washed in distilled water for 2 min. Finally, the sections were stained with hematoxylin for 5 min and eosin for 2 min, and observed under a microscope.

### 2.11. Detection of Ferroptosis-Related Factors—Fe^2+^, ROS

The levels of ROS and Fe^2+^ in the ischemic tissue were quantified using the reactive oxygen species detection kit (E004-1-1, Jiancheng, Nanjing, China) and ferrous ion detection kit (A039-2-1, Jiancheng, Nanjing, China). All procedures were strictly followed according to the instructions, and the fluorescence intensity was measured using a microplate reader at the corresponding wavelength. Simultaneously, the protein concentration of the tissue samples was determined using the BCA reagent kit (Beyotime, P0010S, Nanjing, China). The results were normalized and expressed as unit fluorescence intensity per milligram of protein.

### 2.12. RT-qPCR Verification

Total RNA was extracted from rat ischemic brain tissues using TRIzol reagent (Thermo Fisher Scientific, Waltham, MA, USA) according to the manufacturer’s instructions. mRNA was reverse transcribed to cDNA using the Revert Aid First Strand cDNA Synthesis Kit (Thermo Fisher Scientific). qPCR was performed using PowerUp SYBR Green Premix (Thermo Fisher Scientific), with gene expression normalized to Hprt1 expression. Relative changes in mRNA levels among groups were quantified using the 2^−△△Ct^ method. Primers are listed in Table 1.

### 2.13. Statistical Analysis

Statistical analysis was performed using R (version 4.3.0). All tests were two-sided, and *p*-values < 0.05 were considered statistically significant.

## 3. Results

### 3.1. Screening of Ferroptosis-Related Key Genes and Prediction of Diagnostic Efficacy

#### 3.1.1. Quality Control

Single-cell data files for GSE227651 were downloaded from the NCBI GEO public database. Considering the quality of data from multiple samples, cells with fewer than 200 captured genes were filtered out using the formula: (nFeature_RNA > 200 & percent.mt ≤ median + 3MAD & nFeature_RNA ≤ median + 3MAD & nCount_RNA ≤ median + 3MAD & percent.ribo ≤ median + 3MAD). Here, nFeature_RNA represents the number of genes, nCount_RNA denotes total UMI counts, percent.mt indicates the mitochondrial read percentage, and percent.ribo represents the ribosomal read percentage. Doublet cells were then removed using the DoubletFinder v2.0.6package, retaining 43,664 cells. Post-filtering violin plots and scatter plots are shown (Supplementary Figure S1A,B). The study also displays gene expression across samples and highlights the top ten genes with the highest normalized variance (Supplementary Figure S1C).

#### 3.1.2. Data Normalization and Cell Annotation

**Figure 2:**
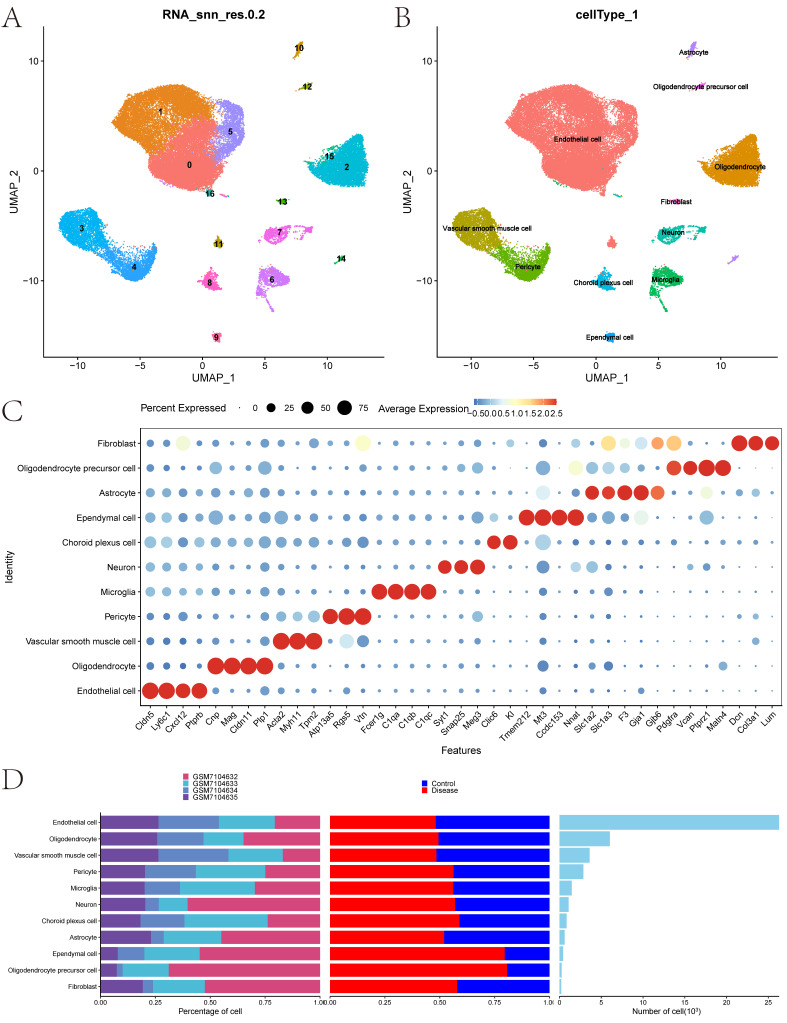
Data Processing and Cell Annotation. (**A**): UMAP analysis for dimensionality reduction and visualization of cell clustering. (**B**): Annotation of cell subtypes categorized into specific classes. (**C**): Bubble chart depicting classical markers for each identified cell subtype. (**D**): Histogram illustrating the proportion of each cell subtype across samples.

Data were sequentially standardized, normalized, and processed through PCA, harmony, and UMAP analyses (Supplementary Figures S1D–F and S2A). Each subtype was annotated, assigning clusters to eleven cell categories: Endothelial cell, Oligodendrocyte, Vascular smooth muscle cell, Pericyte, Microglia, Neuron, Choroid plexus cell, Ependymal cell, Astrocyte, Oligodendrocyte precursor cell, and Fibroblast (Figure 2B). Classic biomarkers for these eleven cells were displayed in a bubble chart (Figure 2C) along with a histogram showing the cell proportions for each group (Figure 2D).

#### 3.1.3. Quantification of Immune Cell Ferroptosis Score and GO and KEGG Functional Annotation

**Figure 3:**
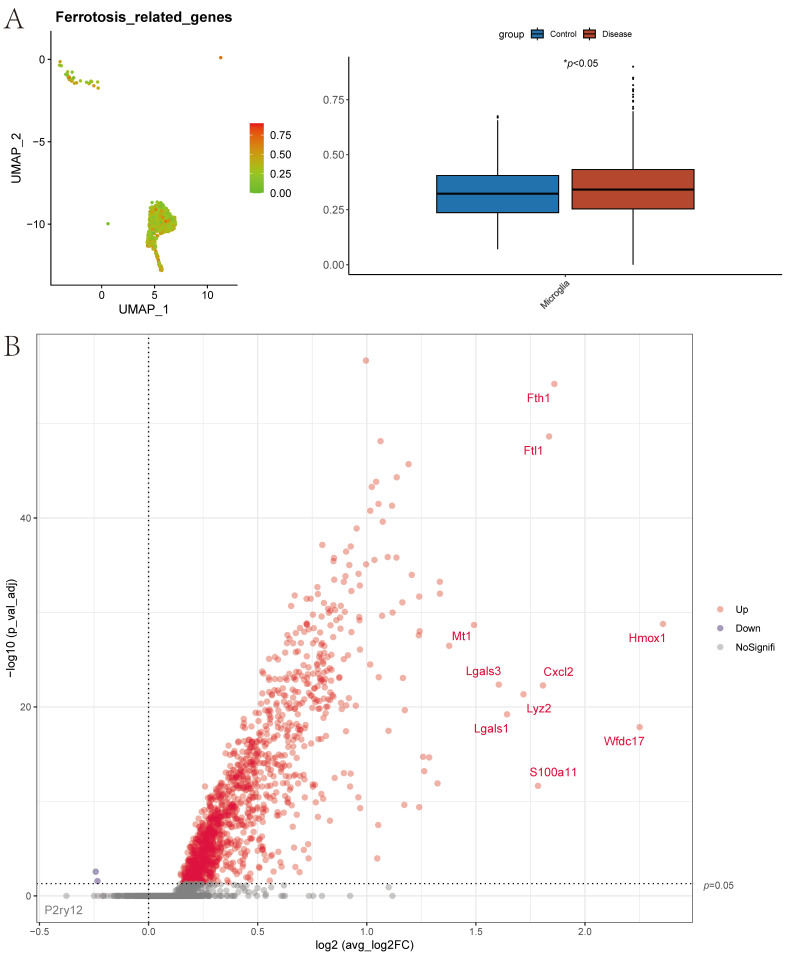
Single-Cell Level Ferroptosis Scoring and Differential Gene Screening. (**A**): Ferroptosis scores at the single-cell level(* *p* < 0.05). (**B**): Volcano plot depicting 303 differentially expressed genes identified through ferroptosis scoring analysis.

**Figure 4:**
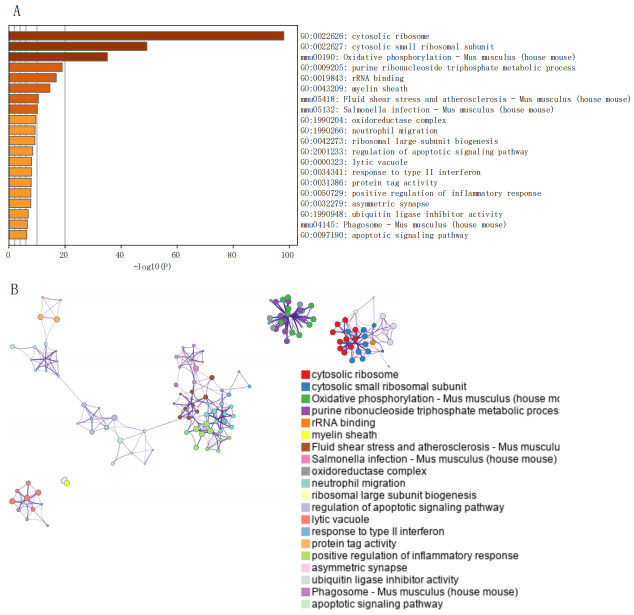
Enrichment Pathways of 303 Differential Genes. (**A**): GO enrichment analysis. (**B**): KEGG enrichment analysis.

The ferroptosis gene set, comprising 60 genes related to ferroptosis, was obtained from previous research [22]. At the single-cell level, ferroptosis scores were assessed using the AUCell function. Analysis revealed significant differences in scores between control and disease groups in Microglia (Figure 3A). Differential gene analysis between high and low scoring Microglia groups was performed, filtering genes with *p*-value < 0.05 & logFC > 0.585. A total of 303 differential genes were identified and visualized in a volcano plot (Figure 3B). Pathway analysis of these 303 marker genes in Microglia was conducted using Metascape, revealing significant enrichment in pathways such as cytosolic ribosome, cytosolic small ribosomal subunit, and rRNA binding (Figure 4).

#### 3.1.4. Machine Learning Algorithms to Identify Key Genes

**Figure 5:**
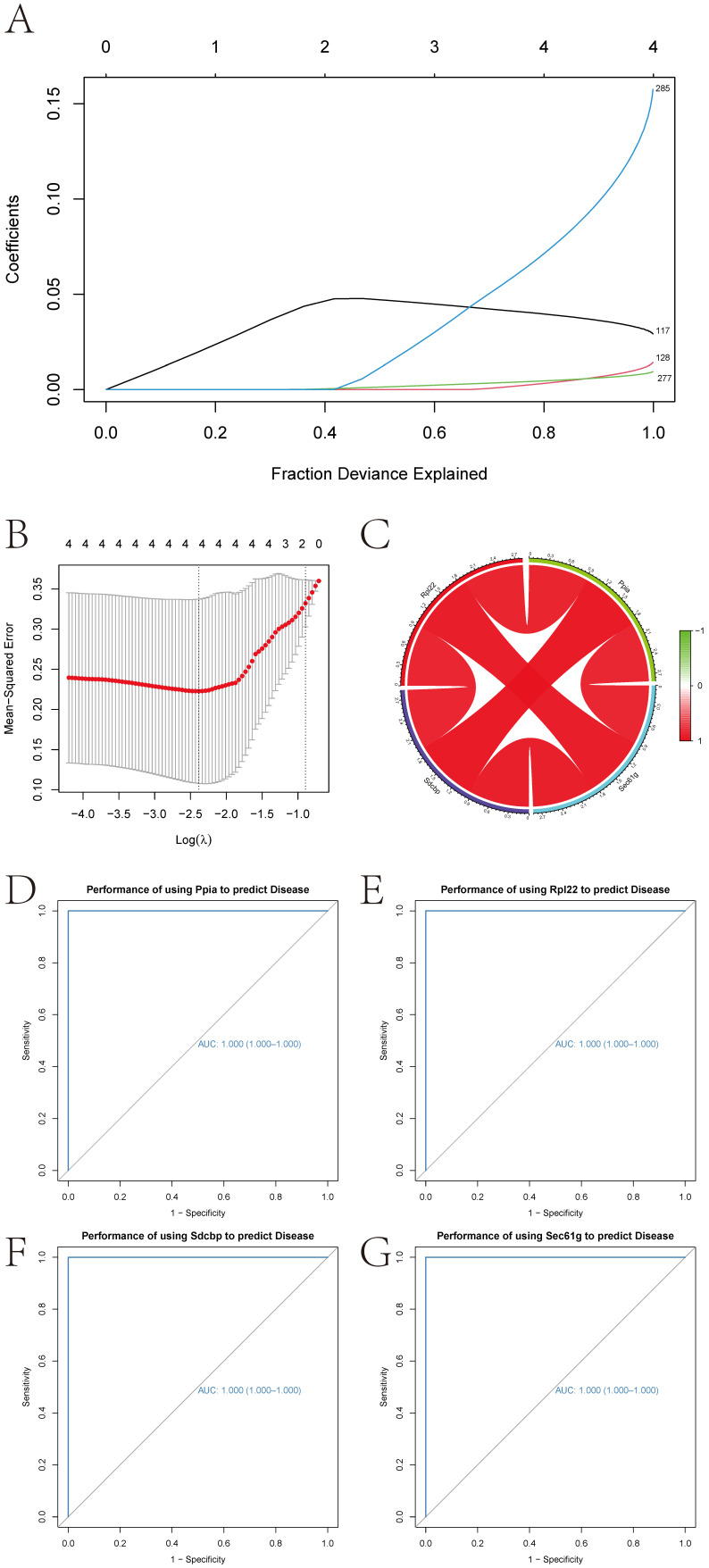
Selection, Interaction, and Diagnostic Efficacy of Key Genes. (**A**,**B**): Selection of four key genes via LASSO regression analysis. (**C**): Interaction network among the four key genes. (**D**–**G**): ROC curves demonstrating the diagnostic efficacy of the four key genes.

Based on 303 marker genes from Microglia, key genes impacting CIRI were identified using lasso regression and random forest, yielding four characteristic genes for CIRI: Sdcbp, Rpl22, Ppia, and Sec61g (Figure 5A,B). These genes are crucial for subsequent research on their roles in CIRI.

#### 3.1.5. Key Gene Interaction Networks and ROC Curves

Interaction networks for the four key genes were analyzed using correlation analysis, illustrated in a circular graph format (Figure 5C). The diagnostic efficacy of these genes was evaluated using ROC curves, with AUC values indicating predictive performance; all four genes demonstrated AUC values of 1, suggesting excellent predictive power for disease progression (Figure 5D–G).

### 3.2. Verification of the Role of Key Genes in CIRI Through In Vivo Experiments

#### 3.2.1. Neurological Deficit Assessment and Tissue Damage Observation in MCAO/R Rats

**Figure 6:**
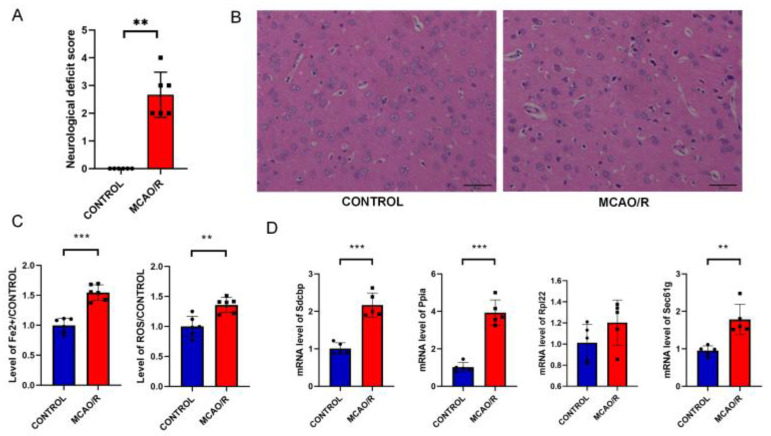
Validation of Animal Experiments. (**A**): Neurological deficit scoring in MCAO/R rats (*n* = 6, ** *p* < 0.01 vs. CONTROL). (**B**): Observation of tissue damage via H&E staining (*n* = 3, magnification 200×). (**C**): Level of Fe^2+^, ROS in MCAO/R rats (*n* = 6, ** *p* < 0.01, *** *p* < 0.001 vs. CONTROL) (**D**): Expression of key genes in MCAO/R rats (*n* = 5, **p < 0.01, *** *p* <0.001 vs. CONTROL).

Zea Longa neurological injury scores indicated no injuries in the CONTROL group with a score of 0. In contrast, scores significantly increased in the MCAO/R group, indicating substantial neurological damage (Figure 6A). H&E staining showed orderly and tightly arranged cells in the CONTROL group, with clear nucleoli indicating normal cell morphology. Compared to the CONTROL group, cells in the MCAO/R group were loosely arranged with shrunken, deeply stained nuclei and extensive vacuolation around the nucleus (Figure 6B), confirming significant brain damage in MCAO/R rats.

#### 3.2.2. Ferroptosis in MCAO Rats

Using ROS and Fe^2+^ detection kits, this study found that MCAO/R rats exhibited Fe^2+^ overload and ROS accumulation, indicating ferroptosis in the rat brain after MCAO/R (Figure 6C).

#### 3.2.3. Expression of Key Genes in MCAO/R Rats

RT-qPCR was used to validate the expression of key genes, revealing significant upregulation of Sdcbp, Ppia, and Sec61g in MCAO/R rats compared to the CONTROL group, while Rpl22 showed no significant change (Figure 6D). This suggests that Sdcbp, Ppia, and Sec61g are associated with ferroptosis in IS injury.

### 3.3. Key Genes in Immune Infiltration and Associated Pathways

#### 3.3.1. Immune Infiltration and Immune Regulatory Factors

**Figure 7:**
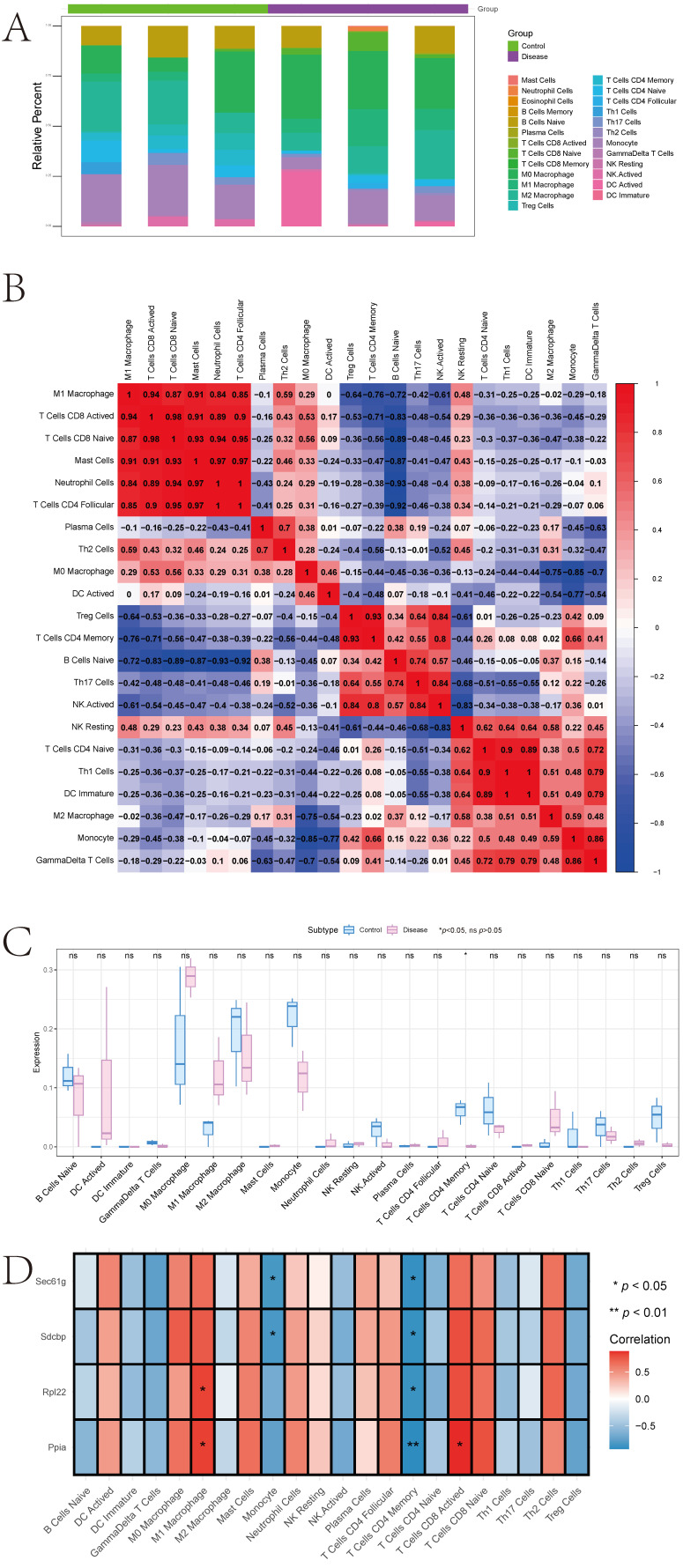
Immune Infiltration and the Association of Key Genes with Immune Cells. (**A**,**B**): Distribution of immune infiltration levels and correlations among immune cells. (**C**): Differences in immune cell levels between control and disease groups. (**D**): Correlations between key genes and immune cells.

**Figure 8:**
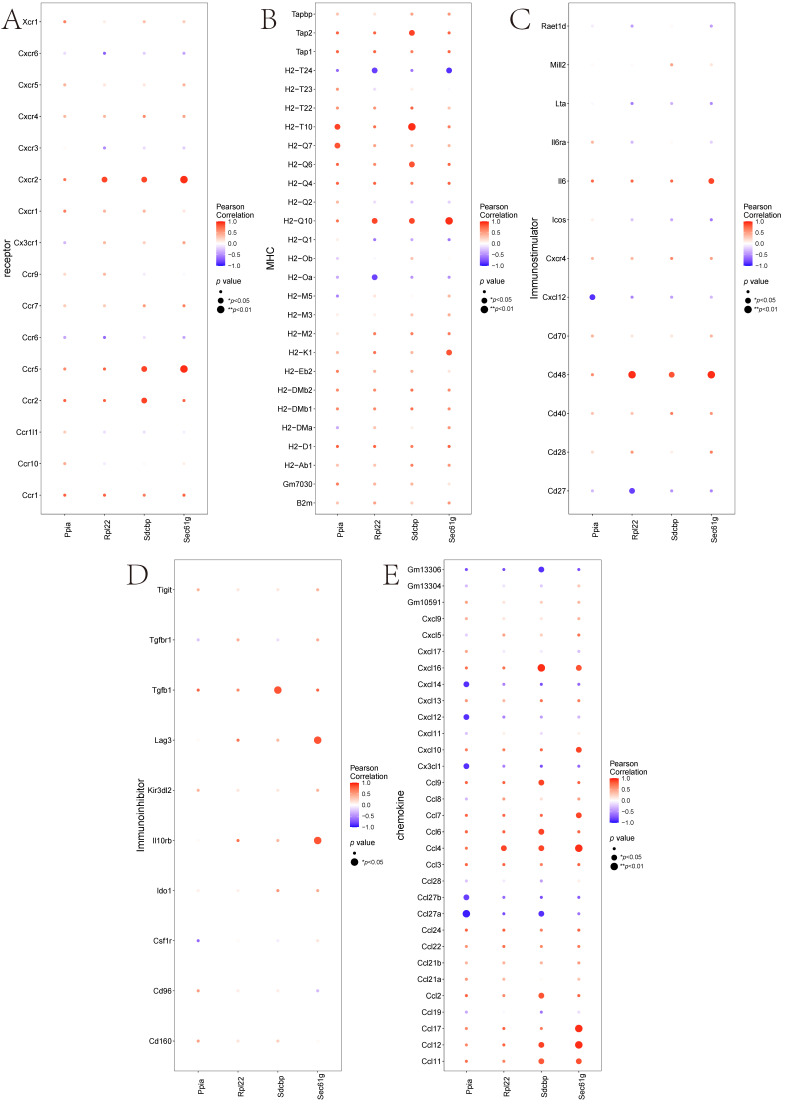
Correlations Between Key Genes and Various Immune Factors. (**A**–**E**): Correlations of key genes with receptors, major histocompatibility complex components, immune stimulatory factors, immune inhibitory factors, and chemokines, respectively.

The microenvironment, composed of fibroblasts, immune cells, extracellular matrix, various growth factors, inflammatory factors, and specific physicochemical characteristics, significantly influences disease diagnostics, survival outcomes, and clinical treatment sensitivity. Immune infiltration levels and immune cell correlations are presented in different formats (Figure 7A,B), with significant differences in T Cells CD4 Memory levels between groups (Figure 7C). Further analysis explored relationships between key genes and immune cells, revealing significant correlations between Sdcbp and T Cells CD4 Memory and Monocytes, Rpl22 and M1 Macrophages, and negative correlations with T Cells CD4 Memory. Ppia showed significant positive correlations with T Cells CD8 Active and M1 Macrophages, and negative correlations with T Cells CD4 Memory. Sec61g was significantly negatively correlated with T Cells CD4 Memory and Monocytes (Figure 7D). Additionally, analyses of key genes’ correlations with different immune factors including receptors, major histocompatibility complex, immune stimulatory and inhibitory factors, and chemokines indicated that key genes are closely associated with immune cell infiltration levels and play significant roles in the immune microenvironment (Figure 8A–E).

#### 3.3.2. Transcription Factor Regulatory Networks

**Figure 9:**
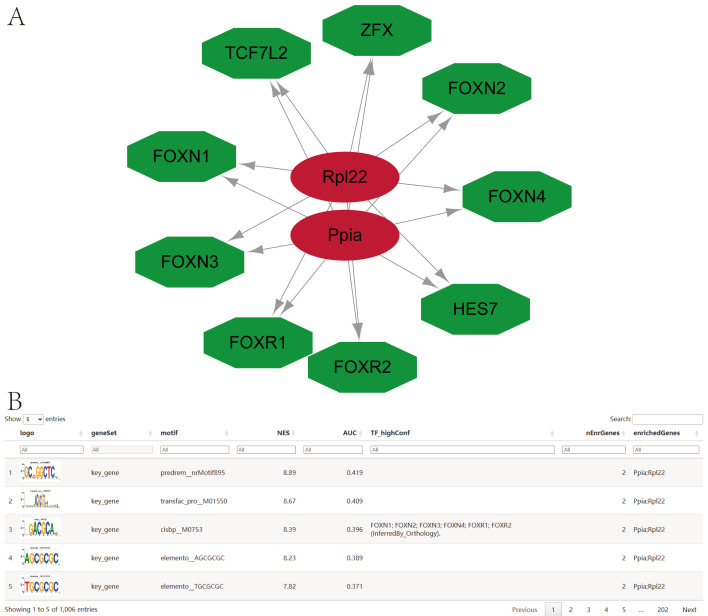
Enrichment of Motifs and Corresponding Transcription Factors in Ppia and Rpl22. (**A**): Common transcription factor regulatory network. (**B**): Enrichment of Motifs.

Key genes were analyzed for regulation by multiple transcription factors, with enrichment analyses performed using cumulative recovery curves. Motif-TF annotations and key gene selection analysis identified the motif with the highest normalized enrichment score (NES: 8.89) as predrem__nrMotif895. All enriched motifs and corresponding transcription factors for the key genes were displayed (Figure 9A,B).

#### 3.3.3. Expression of Key Genes in Single Cells

**Figure 10:**
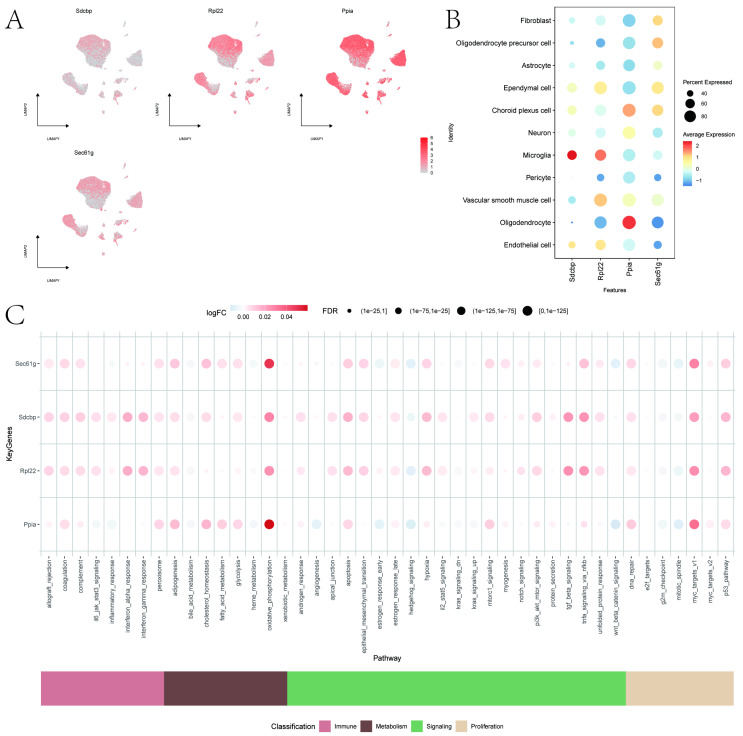
Expression of Key Genes in Single Cells and Immune Metabolic Pathways. (**A**,**B**): Expression of key genes in single cells. (**C**): Expression of key genes in immune metabolic pathways.

Key gene expressions in single cells were visualized using Dotplot and FeaturePlot functions in the Seurat v4package (Figure 10A,B), showing that Sdcbp and Rpl22 were most abundantly expressed in Microglia, and Ppia was most abundant in Oligodendrocytes, while Sec61g showed lower overall expression. AUCell was used to quantitatively score genes related to immune metabolic pathways in single cells, with bubble charts displaying expression variations in key genes in pathways such as TNFA_SIGNALING_VIA_NFKB and OXIDATIVE_PHOSPHORYLATION (Figure 10C). Disease regulatory genes were obtained from the GeneCards database (https://www.genecards.org/, accessed on 18 September 2024), with the top-ranked genes by Relevance score selected for co-expression network analysis with key genes (Supplementary Figures S2–S5).

## 4. Discussion

CIRI is a critical factor in IS damage. Early diagnosis, detection, and timely treatment are essential for reducing these harmful effects. Although the pathophysiology of CIRI is complex, involving oxidative stress, cell apoptosis, immune inflammation, and neurotoxicity from amino acids, with mechanisms that intricately interplay, bioinformatics can help understand the molecular mechanisms of CIRI and assist in identifying potential therapeutic targets. Previous studies have highlighted the role of ferroptosis in CIRI [23,24,25], yet research connecting ferroptosis with immune infiltration in the context of CIRI is limited. Therefore, this study aims to identify key ferroptosis-related genes in CIRI using bioinformatics and analyze their links with immune infiltration to find targeted therapeutic interventions.

Initially, four key ferroptosis genes were identified. After quality control, data normalization, and cell annotation of the single-cell data GSE227651, ferroptosis scores were evaluated at the single-cell level across 11 cell categories. Significant differences in Microglia ferroptosis scores were noted between control and MCAO/R groups. Existing studies suggest diverse phenotypic changes in neurons, astrocytes, microglia, endothelial cells, and pericytes in specific cell types post-IS, with microglia constituting a significant proportion of cells post-transient MCAO [26,27]. This underscores the critical role of Microglia in CIRI, supporting our hypothesis that ferroptosis could be a contributing factor. Subsequent differential analysis identified 303 ferroptosis-related differential genes, primarily enriched in pathways such as cytosolic ribosome, small ribosomal subunit, and rRNA binding. Furthermore, Lasso regression identified four characteristic genes post-CIRI ferroptosis: Sdcbp, Rpl22, Ppia, and Sec61g, with ROC curves confirming their strong diagnostic value. Similarly, Liu et al. [28] using GEO microarray data, found that HMOX1, STAT3, CYBB, and TLR4 could serve as therapeutic targets for ischemic ferroptosis, verified through animal experiments. Our study employed a similar analytical approach, initially discovering four ferroptosis-related genes with promising diagnostic potential.

Additionally, we confirmed the role of these key genes in CIRI. The Sdcbp gene, located on chromosome 8q12, encodes the Syntenin-1 protein, which influences receptor protein transport, cytoskeletal formation, exosome production, and activation of certain signaling pathways, thereby regulating various physiological and pathological processes [29,30,31,32,33,34]. Transcriptomic data and single-cell RNA sequencing have shown significant upregulation of Sdcbp in whole blood cells of acute myocardial infarction patients, indicating its potential as a prognostic factor for post-myocardial infarction heart failure [35]. Rpl22, an RNA-binding protein, is interestingly linked to the degradation of p53 [36], which promotes ferroptosis [37], suggesting a potential connection between Rpl22 and ferroptosis. Ppia, also known as Cyclophilin A (Cyp A), is involved in oxidative stress-mediated cardiovascular disorders such as heart failure [38], atherosclerosis [39], and aortic dissection [40]. Peter Seizer et al. found that Cyp A expression was elevated in acute myocardial infarction patients and mice, with Cyp A deficiency improving infarct size and contractile function on day 7 post-I/R injury in mice [41]. Sec61g, a component of the Sec61 translocon complex, facilitates the translocation of secretory and membrane proteins through the endoplasmic reticulum [42], primarily studied in the context of cancer. Our study constructed a rat MCAO model using the thread embolism method, with neurological deficit scoring and H&E staining showing significant neurological damage in the MCAO/R group compared to controls. Additionally, Fe^2+^ overload and ROS accumulation were observed, indicating the possibility of ferroptosis, but further confirmation is still required with ferroptosis inhibitors such as liproxstatin-1 and ferrostatin-1. Furthermore, RT-qPCR validation of the four key genes post-I/R revealed significant upregulation of Sdcbp, Ppia, and Sec61g, with no significant change in Rpl22 expression, Which may stem from inherent differences between single-cell datasets and whole-tissue samples. These results indicate significant brain damage after CIRI, and ferroptosis may be involved. Moreover, the key genes Sdcbp, Ppia, and Sec61g may be associated with ferroptosis in CIRI, while insufficient evidence supports a role for Rpl22.

Finally, through correlation and enrichment analyses, we further explored the links between key genes, immune infiltration, and their pathways. Acute strokes often result in the infiltration of peripheral immune cells, T cells, and B cells into the ischemic brain parenchyma, typically considered a detrimental exacerbation of injury [43]. Our study found significant positive or negative associations of the four key genes with M1 macrophages, CD4+ T cells, CD8+ T cells, and monocytes, as well as significant correlations with immune factors like Cxcr2, Ccl27a, Ccl4, H2-T10, and CD48, suggesting that these key genes may indicate the occurrence of immune infiltration. Previous research has shown high expression of the Sdcbp gene in lung adenocarcinoma and squamous cell carcinoma significantly correlated with elevated markers for CD4+ T cells, CD8+ T cells, and macrophages [44]. Ppia promotes the accumulation of neutrophils and macrophages and renal injury in an acute kidney injury model [45], consistent with our findings. However, no studies have linked Rpl22 and Sec61g with immune infiltration. Additionally, we discovered that Rpl22 and Ppia are co-regulated by nine transcription factors including FOXN1 and FOXR1, indicating a common mechanism of action. Our study also found that Sdcbp and Rpl22 were most abundantly expressed in Microglia, with Ppia highly expressed in Oligodendrocytes, while Sec61g showed lower overall expression. Interestingly, we previously found no expression changes in Rpl22 at the RNA level throughout the whole tissue. Therefore, we speculate that Rpl22 may be highly expressed in a specific immune cell population, and its expression products play an important role in immune regulation, which further enhances our previous explanation. These key genes are active in pathways closely related to I/R injury, including TNFA_SIGNALING_VIA_NFKB, MYC_TARGETS_V1, OXIDATIVE_PHOSPHORYLATION, TGF_BETA_SIGNALING, and REACTIVE_OXIGEN_SPECIES_PATHWAY [46,47,48,49,50]. We also explored the interactions of these four key genes with disease-related genes Glul, Nfe2l2, Bax, Tug1, and Hcrt, aiming to understand the progression of disease. Our research indicates that Sdcbp, Ppia, and Sec61g are associated with ferroptosis and immune cell infiltration after IS, suggesting these genes may serve as potential biomarkers. However, the exact causal relationship between them cannot be confirmed at present.

Despite our study’s analysis at the single-cell level, it has limitations. The animal experiments involved a relatively small sample size; a post hoc power analysis confirmed sufficient power (>80%) for key outcomes with large effect sizes (Neurological scores, Ppia), while indicating moderate power (50–80%) for some gene expression changes (Sdcbp, Sec61g, Rpl22). Additionally, no ferroptosis inhibitor was used in our study, making it impossible to confirm the correlation between key genes and the occurrence of ferroptosis. Nonetheless, we preliminarily screened and validated biomarkers associated with ferroptosis and immune infiltration in I/R injury, emphasizing the significance of our study.

## 5. Conclusions

In summary, through bioinformatics analysis of data from the GEO database, we screened key genes related to ferroptosis in I/R injury and obtained preliminary validation in animal experiments. Importantly, through analyzing the relationships between these key genes and immune cells, immune factors, and immune metabolic pathways, we found that the key genes Sdcbp, Ppia, and Sec61g may be associated with ferroptosis and immune infiltration after I/R injury. We predict that these genes may serve as potential biomarkers for ferroptosis and immune infiltration after ischemia. However, further research is required to confirm these findings. This study hopes to provide insights for the clinical treatment of ischemic stroke.

## Figures and Tables

**Table 1:** Primer Sequences.

Primer Name	Sequence (5′–3′)
Sdcbp-F	GATGCGGGGATTAGGAGAGC
Sdcbp-R	AGGAAGACTGGAAGCGTTCG
Rpl22-F	GCCTGTGAAAAAGCTTGTGG
Rpl22-R	CTGGAGGAACTGCTCAAAATTGG
Ppia-F	GCCAAGACTGAGTGGCTGGAT
Ppia-R	CCACAATGCTCATGCCTTCTTT
Sec61g-F	GGTTCCGTTGGGCTCAATTC
Sec61g-R	GAAGCCGATGAACCCCATGA
Hprt1-F	GTCCCAGCGTCGTGATTAGT
Hprt1-R	CTTGCCGCTGTCTTTTAGGC

## Data Availability

The original contributions presented in this study are included in the manuscript/Supplementary Materials. Further inquiries can be directed at the corresponding authors.

## Supplementary Materials

The following supporting information can be downloaded at: https://www.mdpi.com/article/10.3390/cimb47121066/s1.
