# Supplementary material for: Bioinformatics Identification and Experimental Validation of Ferroptosis- and Immune Infiltration-Associated Biomarkers in Ischemic Stroke
Source: Curr Issues Mol Biol. 2025 Dec 18;47(12):1066. doi: 10.3390/cimb47121066 (PMC12732058; doi:10.3390/cimb47121066)
Supplement: Supplementary file 1 [file cimb-47-01066-s001.zip › cimb-3998931-supplementary.pdf]

Supplementary Materials

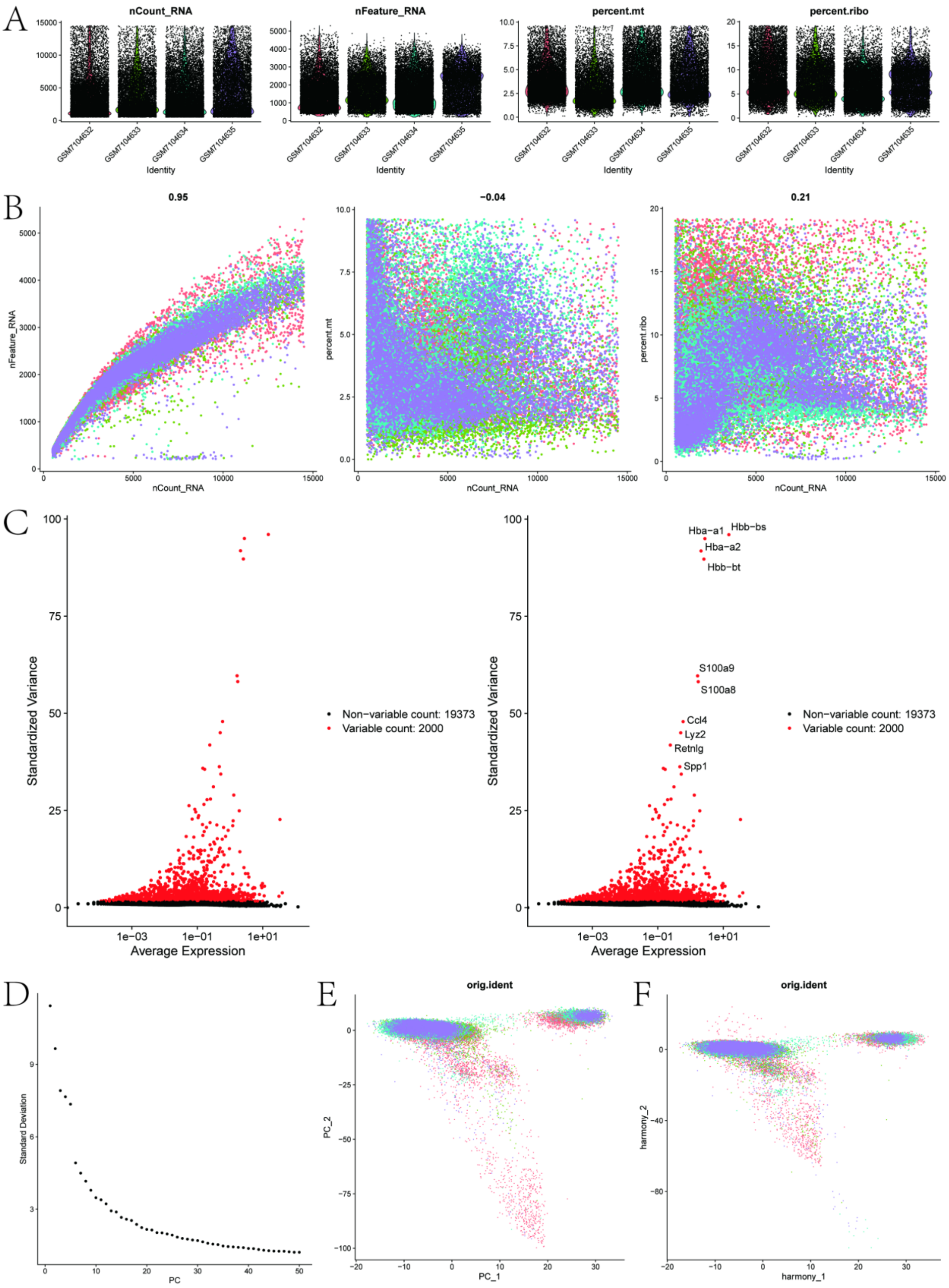

Supplementary Figure S1 Single-Cell Data Quality Control and Normalization A: Violin plots displaying cell metrics post-filtering. B: Scatter plots illustrating cell distribution post-filtering. C: Markers of the top 10 genes with the highest normalized variance. D-F: Data normalization, standardization, PCA, and Harmony processing steps.

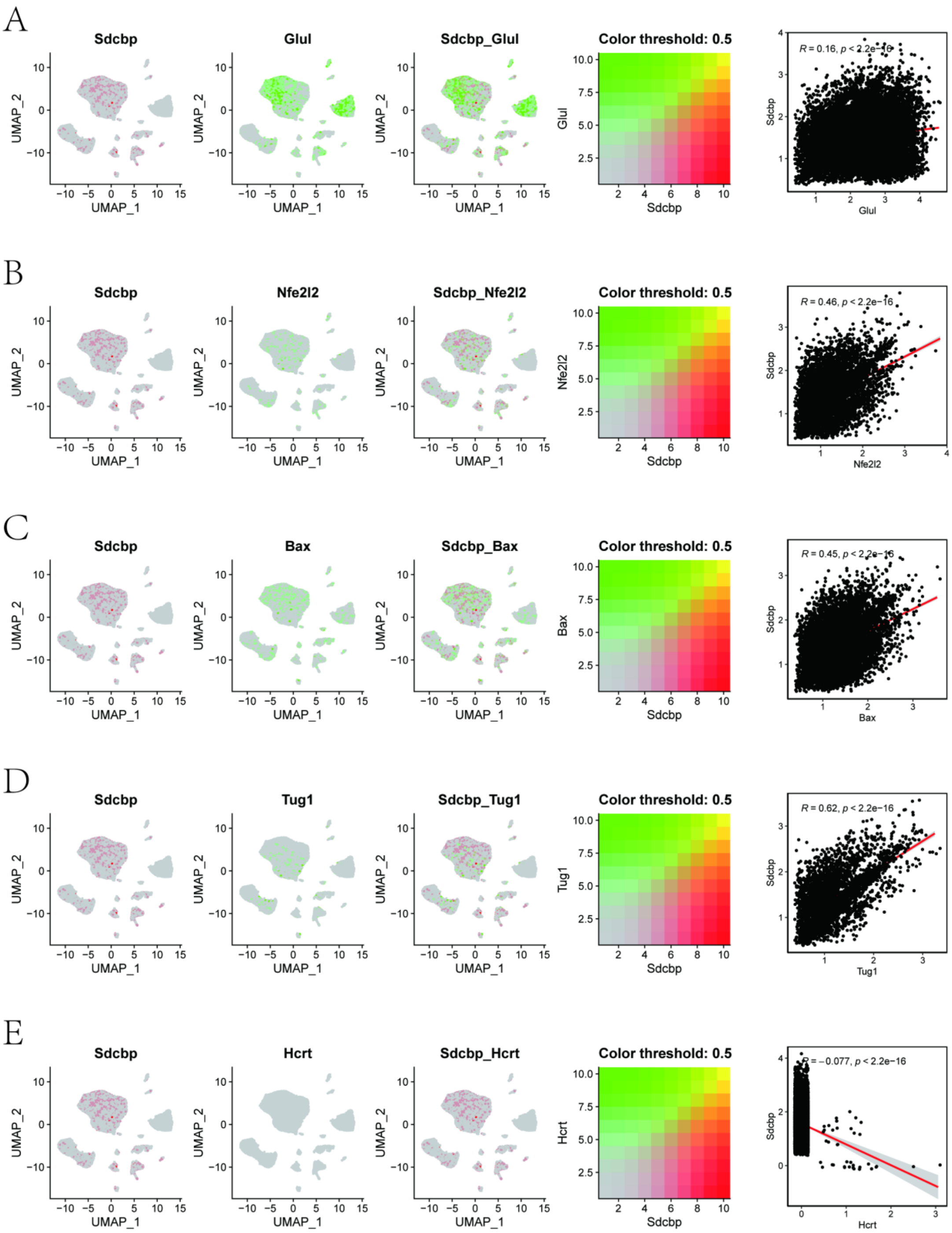

Supplementary Figure S2 Co-expression of Key Gene Sdcbp with Disease Genes A-E: Each panel represents the interaction relationships between the key gene Sdcbp and disease-related genes Glul, Nfe2l2, Bax, Tug1, and Hcrt, respectively.

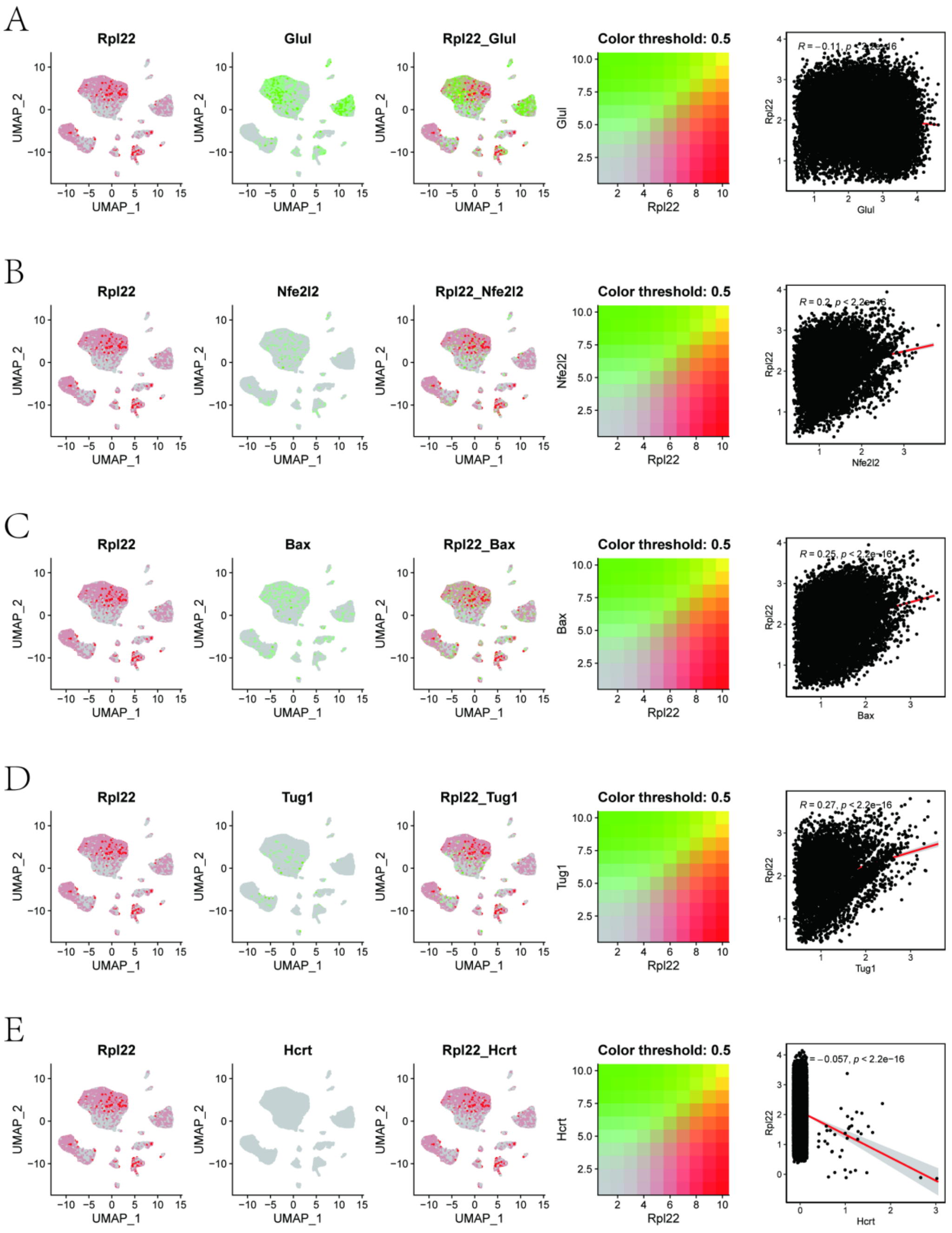

Supplementary Figure S3 Co-expression of Key Gene Rpl22 with Disease Genes A-E: Each panel illustrates the interaction relationships between the key gene Rpl22 and disease-related genes Glul, Nfe2l2, Bax, Tug1, and Hcrt, respectively.

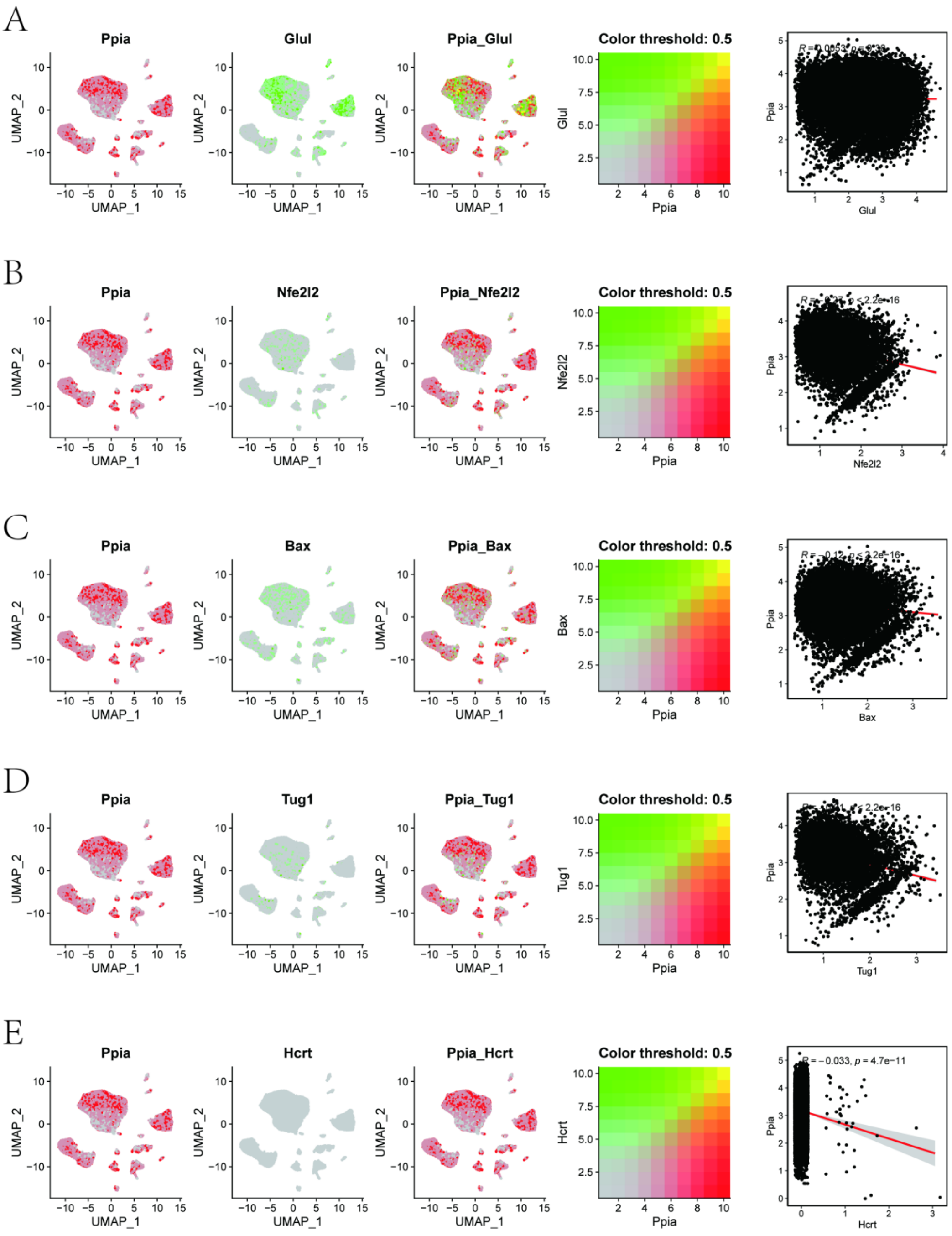

Supplementary Figure S4 Co-expression of Key Gene Ppia with Disease Genes A-E: Each panel depicts the interaction relationships between the key gene Ppia and disease-related genes Glul, Nfe2l2, Bax, Tug1, and Hcrt, respectively.

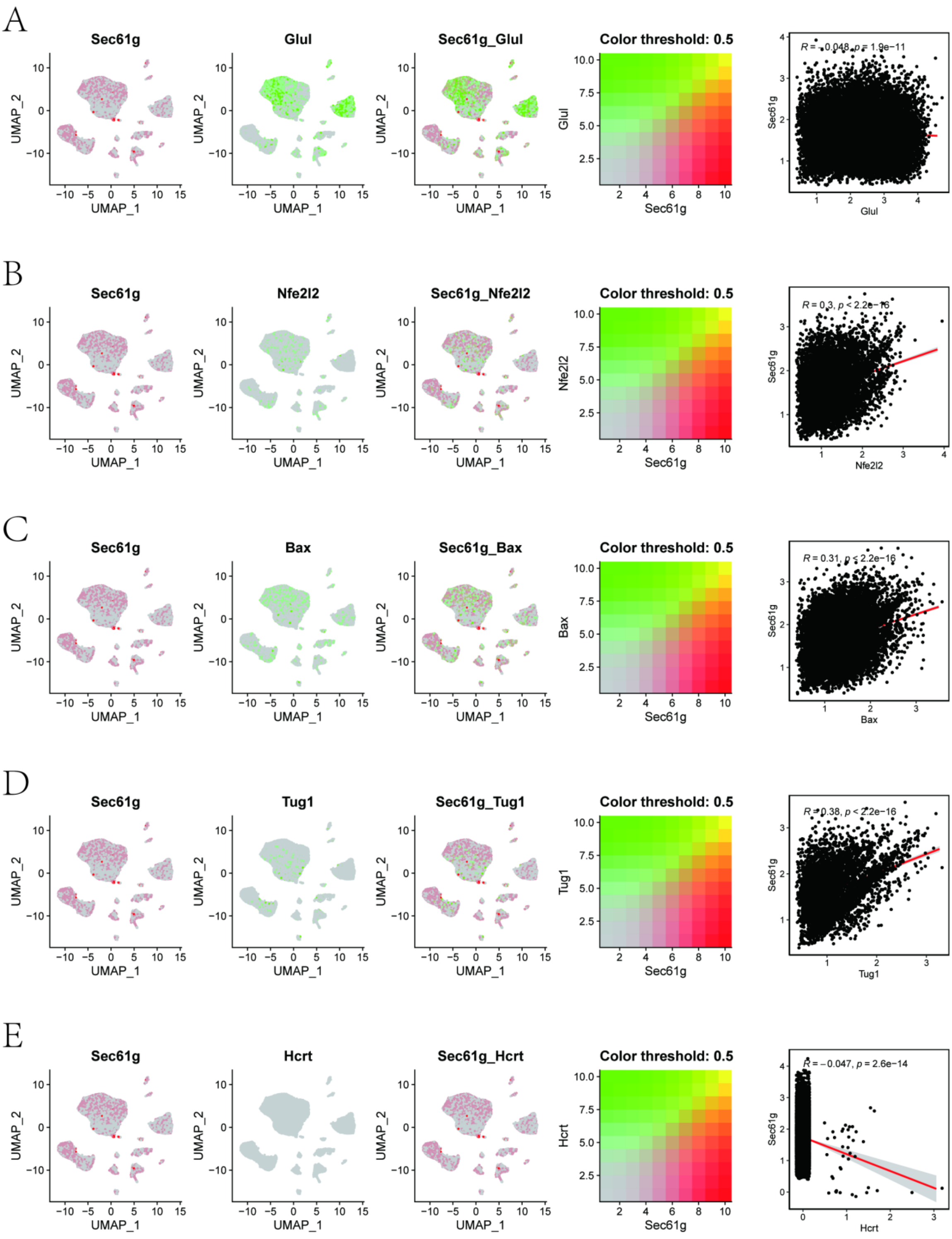

Supplementary Figure S5 Co-expression of Key Gene Sec61g with Disease Genes A-E: Each panel illustrates the interaction relationships between the key gene Sec61g and disease-related genes Glul, Nfe2l2, Bax, Tug1, and Hcrt, respectively.
